# Supplementary material for: Bound-State Breaking and the Importance of Thermal Exchange–Correlation Effects in Warm Dense Hydrogen
Source: J Chem Theory Comput. 2023 Dec 22;20(1):68–78. doi: 10.1021/acs.jctc.3c00934 (PMC10782774; doi:10.1021/acs.jctc.3c00934)
Supplement: Supplementary file 1 — ct3c00934_si_001.pdf [file ct3c00934_si_001.pdf]

Supplemental Material: Bound state breaking and the importance of thermal exchange–correlation effects in warm dense hydrogen

Zhandos Moldabekov,[*,†,‡] Sebastian Schwalbe,[†,‡] Maximilian P. Böhme,[†] Jan Vorberger,[‡] Xuecheng Shao,[¶] Michele Pavanello,[¶] Frank R. Graziani,[‖] and Tobias Dornheim[*,†,‡]

†*Center for Advanced Systems Understanding (CASUS), D-02826 Görlitz, Germany*

‡*Helmholtz-Zentrum Dresden-Rossendorf (HZDR), D-01328 Dresden, Germany*

¶*Department of Chemistry, Rutgers University, Newark, NJ 07102, USA*

§*Department of Physics, Rutgers University, Newark, NJ 07102, USA*

‖*Lawrence Livermore National Laboratory (LLNL), California 94550 Livermore, USA.*

E-mail: z.moldabekov@hzdr.de; t.dornheim@hzdr.de

Ground state bond breaking

Kohn-Sham density functional theory (KS-DFT)[1,2] is the workhorse of ground-state electronic structure investigations given its often suitable accuracy and reasonable computational effort.

However, it is well known that KS-DFT is not able to correctly describe the dissociation of molecules.[3,4] In KS-DFT electrons artificially interact with themselves; this is known as the

self-interaction (SI) error. The Perdew-Zunger self-interaction correction (PZ-SIC)[5] removes the SI with an orbital-by-orbital correction. In contrast to KS-DFT, it delivers an accurate description of the dissociation limit.[3] A novel flavor of PZ-SIC is the Fermi-Löwdin orbital self-interaction correction (FLO-SIC).[6–9]

A clear advantage of FLO-SIC is the method-specific concept of Fermi-orbital descriptors (FODs), i.e., semi-classical electron positions. It has been shown that these FODs, as well as the respective localized FLOs, carry bonding information[10,11] and can be used to analyze, interpret, and guide SIC solutions. Recently, FODs were used to describe bond breaking from an electron perspective for a Diels-Alder reaction.[12]

Thus, FLO-SIC is a promising method to analyze bonding for a given system at $T = 0$ K. To analyse the bond dissociation of the H_2 molecule at $T = 0$ K, we investigated critical points in the total energy, density, and reduced density gradient (RDG) along the dissociation curve to enable a comparison with finite temperature calculations.

Along the bond dissociation, one observes different bond situations. For instance, $d \leq 0.75$Å represents a compressed bond, for $d = 0.75$ Å the molecule is close to its ground state geometry (bonding region), and for $d \geq 0.75$ Å one observes the stretched bond region. At large bond separation one reaches the dissociation limit.

As we will see later, the spin of a system is an important property for bond dissociation for $T = 0$ K ground state calculations. The spin of a system in an unrestricted, open-shell description is given by

$$\text{spin} = N_\alpha - N_\beta \tag{1}$$

with N_σ being the number of electrons in the respective spin channel. In the ground state at $T = 0$ K, the H_2 molecule has a spin of 0 with one electrons being in the α channel and the other electron being in the β channel. If both electrons are in one spin channel the spin of the system would be 2.

The total energy of H_2 has been calculated along the bond dissociation (see Fig. S1) for both possible spin values. Once the H_2 bond breaks, a single electron will move to each

hydrogen nucleus. At infinite bond separation, i.e., the atomic dissociation limit, it does not matter if the electron is in the α or the β spin channel.

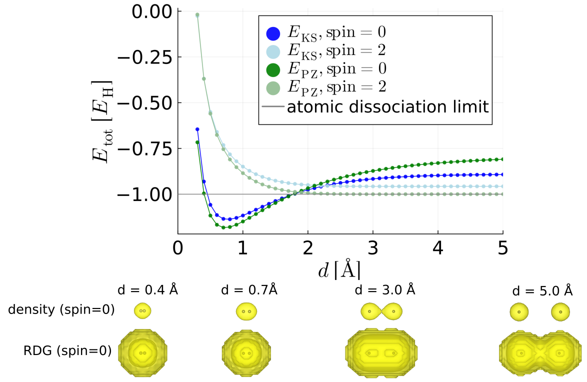

Figure S1: H_2 bond dissociation curve calculated using KS-DFT and FLO-SIC. The bottom line visualize KS densities and RDG for spin=0 for different bonding regions.

FLO-SIC showcases the aforementioned behavior. While the ground state has spin=0, at the bond-breaking distance of $d = 1.9$ Å in the case of FLO-SIC the spin=2 states become energetically preferred. At large separation distances, FLO-SIC energies for spin=2 approach the correct atomic limit, i.e., two times the energy of a hydrogen atom ($\approx -1\ E_{\mathrm{H}}$). Note that FLO-SIC is quasi-exact for the H atom. The accuracy is only limited by the used basis set and numerical quadrature. For the numerical parameters used for our investigations we get a total energy of $-0.499956\ E_{\mathrm{H}}$. In clear contrast, KS-DFT does not give the correct energies at the atomic dissociation limit (see Fig. S1).

We present the RDG for the H atom (see Fig. S2 (a)) and the H_2 molecule ($d = 0.75$ Å, see Fig. S2 (b)). Clearly, there is a distinct peak around $n \approx 0$ for KS-DFT. The same behavior is observed for FLO-SIC (not shown). This can be understood more intuitively from the 3D representation of the RDG. In the compressed bonding and bonding region the RDG has approximately the form of an ellipsoid (see Fig. S1) and in the stretched bonding region the RDG resembles the shape of a dumbbell (see Fig. S1). While the density in those regions resembles an ellipsoid, the RDG has some sort of leveled surface structure. For $d = 5.0$ Å the RDG has the form of two spheres only marginal touching each other, while the density for this values has the form of two separated spheres. For $d \geq 6.0$ Å the RDG

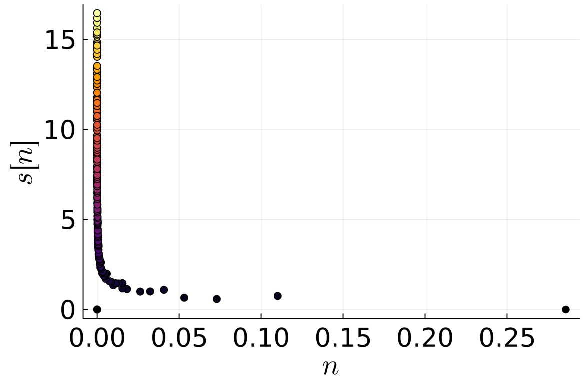

(a) KS-DFT RDG for H atom

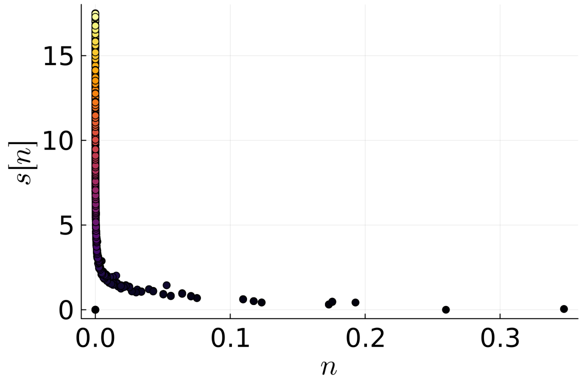

(b) KS-DFT RDG for H_2 ($d = 0.75$ Å)

Figure S2: KS-DFT RDG values for the H atom (a) and the H_2 molecule ($d = 0.75$ Å, bonding region) (b). For both the H atom and H_2 molecule there is a sharp peak at low densities $n \approx 0$.

has also the form of two separated spheres. The general features of $s[n]$ for the bonding region (see Fig. S2 (b)), i.e., the peak of $s[n]$ and the ellipsoid shape of the RDG, match the ones of the warm dense hydrogen system presented in Fig. 2 of the main manuscript for $r_s = 4$. The dumbbell shape of the RDG in the stretched bond region match the H_2 feature presented in Fig. 2 of the main manuscript for $r_s = 2$. However, while there is a distinct peak of $s[n]$ in the ground state stretched bond region, no such feature is present in the warm dense hydrogen system of $r_s = 2$. In the ground state atomic dissociation limit the RDG resembles separated spheres, which can be compared to the separated entities presented in Fig. 2 of the main manuscript for $r_s = 1$.

We monitored the RDG for various bond lengths. For all four bonding regions, i.e., the compressed bonding-, the bonding-, the stretched bonding-, and the bond dissociation-region, the distinct peak of $s[n]$ around $n \approx 0$ is present. For small bonding distances $d \leq 1$ Å the RDG reaches its highest values (see Fig. S3 (a)).

Additionally, we investigated $S[\alpha]/S[0]$ with KS-DFT and FLO-SIC along the bond dissociation curve. For all calculated bond lengths $S[\alpha]/S[0]$ can be fitted with $ae^{-b\alpha}$ (see Fig. S3 (b)). For $\alpha = -1$, $S[\alpha]/S[0]$ reaches its maxima and exponentially tends to zero afterwards. Thus in KS-DFT and FLO-SIC, the features presented for warm dense hydrogen

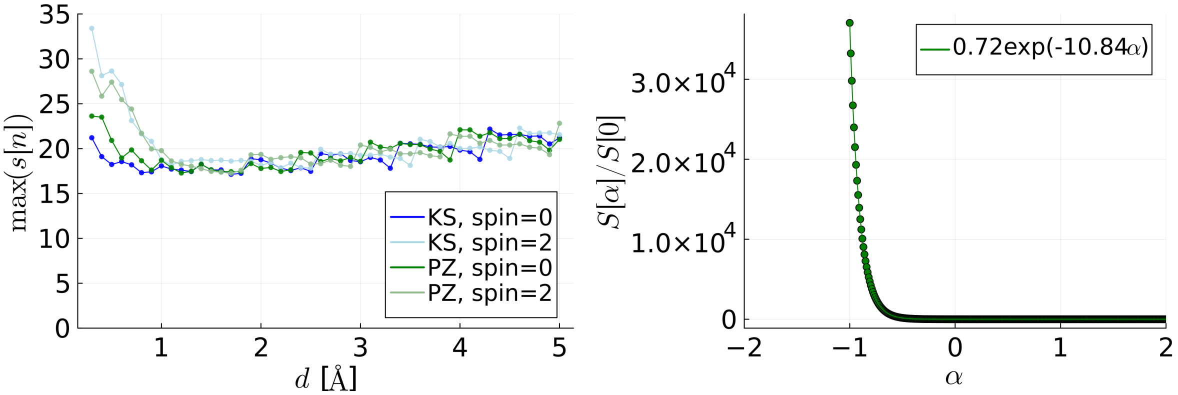

(a) Maxima of RDG for H_2

(b) Generalised dimensionless RDG for H_2 ($d = 0.75$ Å)

Figure S3: Maxima of RDG for various bond lengths (a), and KS-DFT $S[\alpha]/S[0]$ for $d = 0.75$ Å (b). The sharp peak at low densities $n \approx 0$ never smears out in ground state KS-DFT and FLO-SIC for spin=0 and spin=2. This is in clear contrast to the finite temperature calculations presented in the main manuscript. For all investigated bond lengths $S[\alpha]/S[0]$ (see (b) for $d = 0.75$ Å) can be approximated with $ae^{-b\alpha}$. For a bond length of $d = 0.75$ Å, we find $0.72\exp(-10.84\alpha)$. The fit gives a coefficient of determination of $R^2 = 1$.

in the main manuscript (see Fig. 4) are not present for the ground state bond dissociation of the H_2 molecule.

Simulation details for ground state calculations

The all-electron Gaussian-type orbital (GTO) electron structure code CHILLI.JL[12,13] was utilized to perform KS-DFT and FLO-SIC calculations.

We used the local density approximation in form of the LDA-VWN[14] exchange-correlation functional. For the calculations the cc-pVQZ basis set and a numerical quadrature of (150,302) was applied.

Reference calculations have been performed with PySCF.[15] The mean absolute error (MAE) for the total energy of both codes is $4.56 \cdot 10^{-5}$ E_{H}. This indicates a sufficient reproducibility of the presented results for the used numerical parameter space. The quality of the density and RDG has been checked by comparing the presented results to the results

of the plane-wave (PW) electronic structure code EMINUS[16]. For the PW calculations a box size of 20 a_0 and a cutoff energy of $E_{\mathrm{cut}} = 30\ E_{\mathrm{H}}$ was used. The general shapes of the density and RDG agree between the GTO and PW calculations. In addition, the general bond features of the dissociation curve can be reproduced as well. The respective MAE between the GTO CHILLI.JL and PW EMINUS total energies is $3.13 \cdot 10^{-3} E_{\mathrm{H}}$. Given the conceptional difference of the basis sets the agreement is sufficient.

Note that s is sensitive to numerical values of the density close to zero. Therefore we only analyzed $s[n]$ for $n \geq 10^{-5}$ $[1/a_0^3]$.

PIMC and KS-DFT results for different densities at $T = T_F$

To find a correlation between the sign of α_{min} and the breaking of bound states we analysed the RDG data in the range from $r_s = 1$ to $r_s = 4$ with the step 0.5. In Fig. S4, we show the α_{min} dependence on the density parameter computed using PIMC and KS-DFT data in the range from $r_s = 1$ to $r_s = 4$ for the considering configuration of 14 protons. From Fig. S4, we see that the KS-DFT results for α_{min} are in close agreement with the PIMC results at $1.5 \leq r_s \leq 4$ and that the T-LDA based results are in a slightly better agreement with the PIMC data compared to the LDA and PBE based results. The RDG distributions with respect to density computed using the PIMC data are presented in Fig. S5. The RDG distributions computed from KS-DFT simulations using T-LDA are shown in Fig. S6. From Figs S5 and S6, we see that the maximum value of $s[n]$ decreases with the decrease in r_s. By analysing the RDG data and corresponding α_{min} values, we observe that we have $\max(s[n]) > 1$ at $r_s > 2.5$ with $\alpha_{\mathrm{min}} > 0$ and $\max(s[n]) \lesssim 1$ at $r_s \leq 2.5$ with $\alpha_{\mathrm{min}} < 0$.

PIMC results for different temperatures at $r_s = 4$

In Fig. S7, we show the PIMC data for $s[n]$ for four values of temperature at $r_s = 4$, i.e., $T = T_F$, $T = 2T_F$, $T = 4T_F$, and $T = 8T_F$. The increase in the temperature leads to the

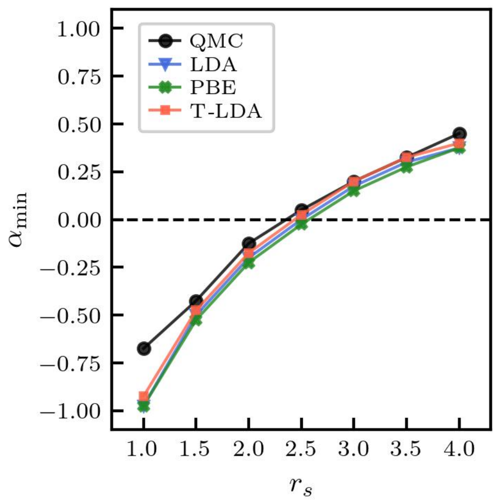

Figure S4: The dependence of the value of α at which $S(\alpha)$ attains its minimum on r_s in the range from $r_s = 1$ to $r_s = 4$ at $T = T_F$.

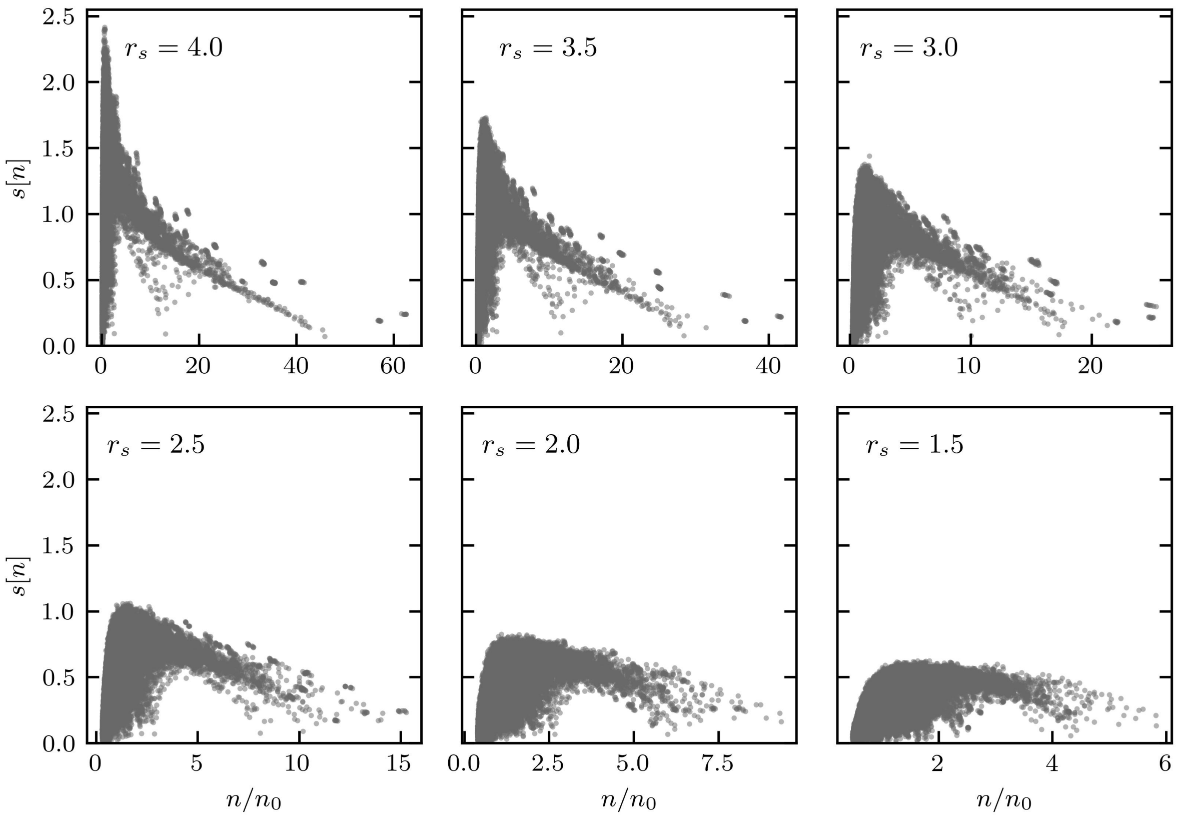

Figure S5: The PIMC results for the distribution of the RDG with respect to density for the warm dense hydrogen with density parameters $r_s = 4.0$, 3.5, 3.0, 2.5, 2.0, 1.5 at the fixed value of the degeneracy parameter $\theta = 1$.

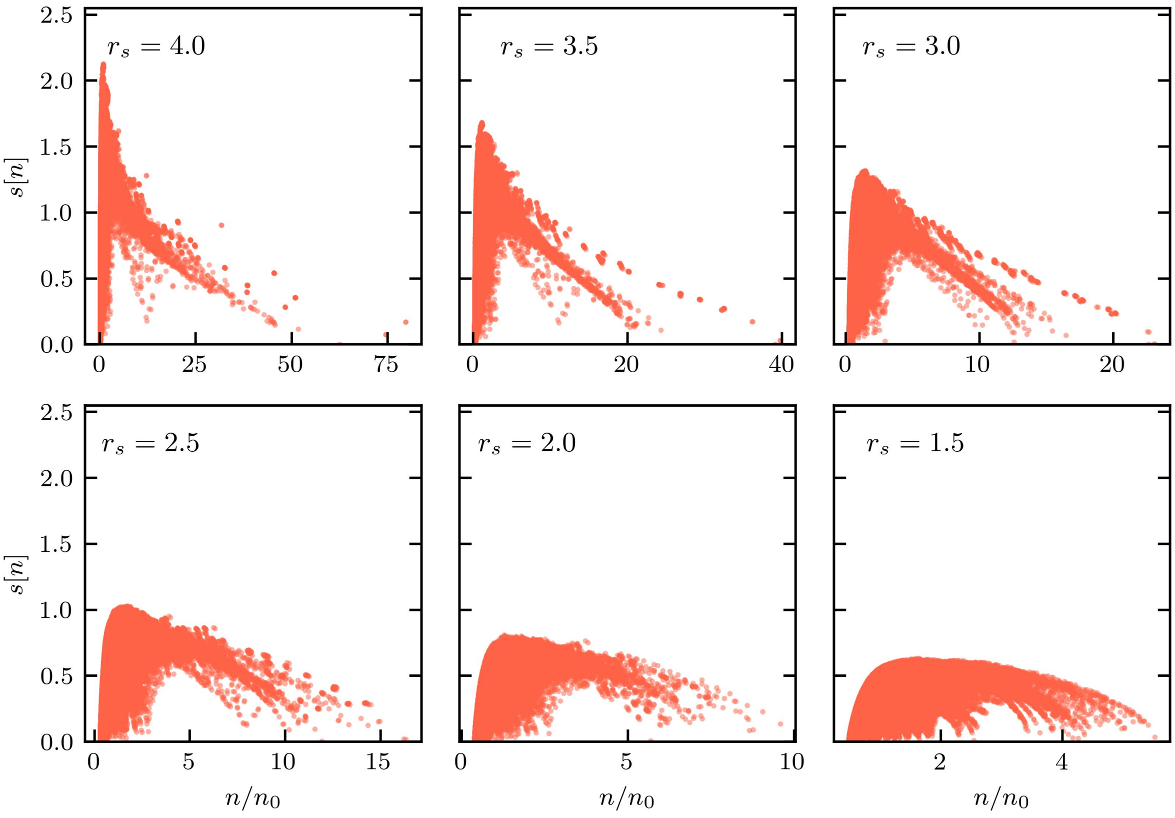

Figure S6: The T-LDA based KS-DFT results for the distribution of the RDG with respect to density for the warm dense hydrogen with density parameters $r_s = 4.0$, 3.5, 3.0, 2.5, 2.0, 1.5 at the fixed value of the degeneracy parameter $\theta = 1$.

decrees of the amplitude of $s[n]$ distribution. This indicates the deterioration of the shell structures of the bound states in hot hydrogen. We note that the ionisation mechanism due to heating at a constant density and due to compression at a constant degeneracy are not equivalent. Particularly, the compression leads to the increase in the Fermi energy and the increase of the effect of the field of other protons on a given molecule (atom). In the case of the heating at a constant temperature, we have only thermal effect induced ionization. In this work, we do not investigate the latter case. Nevertheless, the results presented in Fig. S7 clearly indicate that the RDG based analysis can be used for the investigation of the change in the electronic structure due to heating at a constant value of the density.

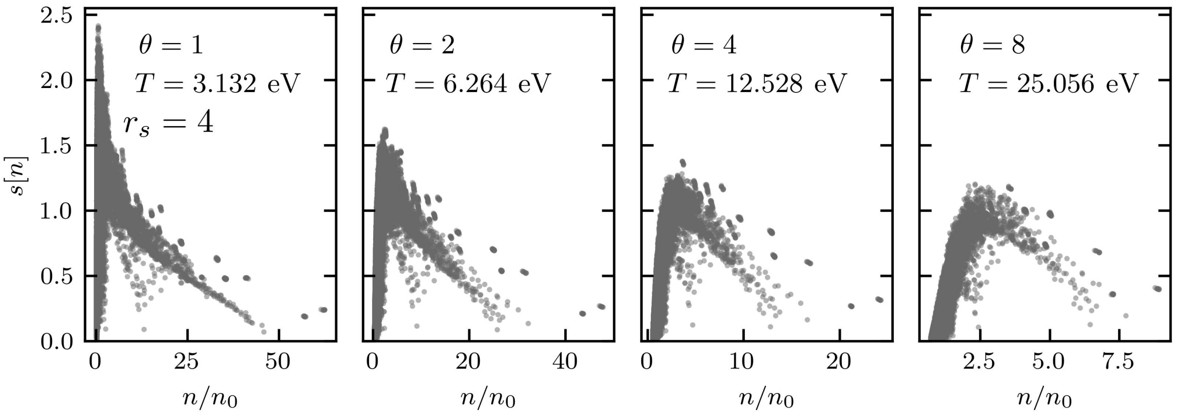

Figure S7: The distribution of the RDG with respect to density for the warm dense hydrogen at different temperatures with the density parameter set to $r_s = 4$.

References

(1) Hohenberg, P.; Kohn, W. Inhomogeneous Electron Gas. Phys. Rev. **1964**, 136, B864.

(2) Kohn, W.; Sham, L. J. Self-Consistent Equations Including Exchange and Correlation Effects. Phys. Rev. **1965**, 140, A1133.

(3) Schwalbe, S.; Hahn, T.; Liebing, S.; Trepte, K.; Kortus, J. Fermi-Löwdin orbital self-interaction corrected density functional theory: Ionization potentials and enthalpies of formation. Journal of computational chemistry **2018**, 39, 2463–2471.

(4) Shahi, C.; Bhattarai, P.; Wagle, K.; Santra, B.; Schwalbe, S.; Hahn, T.; Kortus, J.; Jackson, K. A.; Peralta, J. E.; Trepte, K.; others Stretched or noded orbital densities and self-interaction correction in density functional theory. The Journal of Chemical Physics **2019**, 150, 174102.

(5) Perdew, J. P.; Zunger, A. Self-interaction correction to density-functional approximations for many-electron systems. Phys. Rev. B **1981**, 23, 5048.

(6) Pederson, M. R.; Ruzsinszky, A.; Perdew, J. P. Communication: Self-interaction cor-

rection with unitary invariance in density functional theory. J. Chem. Phys. **2014**, 140, 121103.

(7) Pederson, M. R. Fermi orbital derivatives in self-interaction corrected density functional theory: Applications to closed shell atoms. J. Chem. Phys. **2015**, 142, 064112.

(8) Pederson, M. R.; Baruah, T. Advances In Atomic, Molecular, and Optical Physics; Elsevier, 2015; pp 153–180.

(9) Yang, Z.; Pederson, M. R.; Perdew, J. P. Full self-consistency in the Fermi-orbital self-interaction correction. Phys. Rev. A **2017**, 95, 052505.

(10) Schwalbe, S.; Trepte, K.; Fiedler, L.; Johnson, A. I.; Kraus, J.; Hahn, T.; Peralta, J. E.; Jackson, K. A.; Kortus, J. Interpretation and Automatic Generation of Fermi-Orbital Descriptors. J. Comput. Chem. **2019**, 40, 2843–2857.

(11) Trepte, K.; Schwalbe, S.; Liebing, S.; Schulze, W. T.; Kortus, J.; Myneni, H.; Ivanov, A. V.; Lehtola, S. Chemical bonding theories as guides for self-interaction corrected solutions: Multiple local minima and symmetry breaking. J. Chem. Phys. **2021**, 155, 224109.

(12) Schulze, W. T.; Schwalbe, S.; Trepte, K.; Croy, A.; Kortus, J.; Gräfe, S. Bond formation insights into the Diels–Alder reaction: A bond perception and self-interaction perspective. The Journal of Chemical Physics **2023**, 158.

(13) Schwalbe, S.; Trepte, K.; Schulze, W. T. chilli.jl [chilli_jl]. 2023, accessed date 12/01/2023; https://doi.org/10.5281/zenodo.7596230.

(14) Vosko, S. H.; Wilk, L.; Nusair, M. Accurate spin-dependent electron liquid correlation energies for local spin density calculations: a critical analysis. Canadian Journal of Physics **1980**, 58, 1200–1211, tex.eprint: https://doi.org/10.1139/p80-159.

(15) Sun, Q.; others Recent developments in the PySCF program package. J. Chem. Phys. **2020**, 153, 024109.

(16) Schulze, W.; Trepte, K.; Schwalbe, S. wangenau/eminus: v2.4.0. 2023, accessed date 12/01/2023; https://doi.org/10.5281/zenodo.7962087.
